# Changes in the expression level of genes encoding transcription factors and cell wall-related proteins during *Meloidogyne arenaria* infection of maize (*Zea mays*)

**DOI:** 10.1007/s11033-021-06677-3

**Published:** 2021-09-01

**Authors:** Arnika Przybylska, Maciej Spychalski

**Affiliations:** 1 Department of Molecular Biology and Biotechnology, Institute of Plant Protection – National Research Institute, Poznan, Poland; 2 Poznan Science and Technology Park of Adam Mickiewicz University Foundation, Poznan, Poland

**Keywords:** *Meloidogyne arenaria*, *Zea mays*, Transcription factors, Glycine-rich proteins, Plant-RKNs interactions, Nematode infection, Gene expression analysis

## Abstract

**Background:**

*Meloidogyne arenaria* is an economically important root-knot nematode (RKN) species whose hosts include maize (*Zea mays*). The plant response to RKN infection activates many cellular mechanisms, among others, changes in the expression level of genes encoding transcription and elongation factors as well as proteins related to cell wall organization.

**Methods and results:**

This study is aimed at characterization of expression of selected transcription and elongation factors encoding the genes *WRKY53, EF1a,* and *EF1b* as well as the ones encoding two proteins associated with cell wall functioning (glycine-rich RNA-binding protein, *GRP* and polygalacturonase, *PG*) during the maize response to *M. arenaria* infection. The changes in the relative level of expression of genes encoding these proteins were assessed using the reverse transcription-quantitative real-time PCR. The material studied were leaves and root samples collected from four maize varieties showing different susceptibilities toward *M. arenaria* infection, harvested at three different time points. Significant changes in the expression level of *GRP* between susceptible and tolerant varieties were observed.

**Conclusions:**

Results obtained in the study suggest pronounced involvement of glycine-rich RNA-binding protein and *EF1b* in the maize response and resistance to RKN.

## Introduction

Root-knot nematodes (RKNs) include more than 60 described *Meloidogyne* (Tylenchidae: Tylenchus) species [1]. These nematodes are highly polyphagous with a very wide host range, comprising both, mono- and dicotyledonous plants [2]. *Meloidogyne arenaria*, along with *M. incognita*, *M. javanica,* and *M. hapla*, is one of the most economically important RKN species [1]. This species is distributed worldwide on most continents and parasitizes many major food crops and ornamental plants grown in tropical, subtropical, and temperate climates, in fields as well as in greenhouses. One of the main monocotyledonous hosts of *M. arenaria* is maize (*Zea mays*) [3], which is also one of the most important food crops worldwide in human and animal nutrition.

*Meloidogyne arenaria* is invasive in the J2 larvae stadium. To get into the root tissue, RKNs use their hollow mouth stylet and after mechanical penetration inject the cell wall-degrading enzymes, effectors, and other virulence factors into the cell [1]. During the invasion, this nematode induces changes in expression of a broad spectrum of genes engaged in numerous processes, including in wound and defense responses, reorganization of the cell wall, cell cycle and cytoskeleton organization to establish giant cells, which in consequence leads to galls formation [4]. The presence of nematodes is recognized by the plant through the perception of the pathogen-associated molecular patterns (PAMPs) located on their surfaces. After PAMPs recognition by plant cells, the first layer of defense response is induced [5].

Many aspects of plant response to nematode infection are still not fully understood. Analyses are conducted on monocotyledonous as well as on dicotyledonous hosts and some similarities and differences in the response between these two groups have been observed [6]. For instance, some studies conducted on monocotyledonous plants indicated activation of jasmonic acid (JA)- and salicylic acid (SA)- mediated pathways in RKN-plant interactions. In compatible interactions, the expression level of genes encoding proteins associated with these pathways was upregulated only in the early stages of infection and found to be suppressed later on, in contrast to incompatible interactions in which upregulation of these genes was observed in later stages as well [7–9].

The plant response to RKNs is known to involve many proteins, including a number of transcription factors that have been established to play important roles in plant-nematode interactions. Interestingly, some differences between mono- and dicotyledonous hosts' response to nematode infection concerning changes in the expression of genes encoding these proteins have also been observed [10, 11]. One of the frequently analyzed groups of transcription factors (TFs) are those that are crucial in the regulation of gene expression and are often described as down-stream defense-responsive genes engaged in the response to biotic and abiotic stress conditions [12]. WRKY family is a major group of transcription factors that may act as positive or negative regulators of both components of the plant innate immunity: pathogen-associated molecular pattern-triggered immunity (PTI) as well as in effector-triggered immunity (ETI) [13]. The involvement of WRKYs has been also reported in development of the host resistance to RKNs infection [14]. One of the central factors in the WRKY network, regulating among others early senescence in plants, is WRKY53 [15]. This factor has been reported also to take part downstream of salicylic acid and to be negatively regulated by signaling through jasmonic acid and ethylene in *Arabidopsis* [16]. Moreover, WRKY53 is induced in rice by chitin oligosaccharides and stimulates the expression of PR proteins and peroxidases [17]. However, its role in plant response to *Meloidogyne* infection has not been analyzed as yet.

From among TFs, the elongation factor 1 (EF1) has been established to play an important role in many processes in plants. EF1, composed of a G-protein (EF1a) and the guanine–nucleotide exchange factor (EF1b), is involved in many processes in plants, mainly in the regulation, proliferation, and differentiation of cells [18, 19]. EF1a is a multifunctional protein, which catalyzes the binding of aminoacyl tRNA to the acceptor site on the ribosome and is involved in various other cellular processes such as signal transduction or nuclear export of proteins [20, 21]. Moreover, it is a major cytoskeleton-associated protein, binding microtubules and microfilaments showing actin-binding activity [22]. It has been reported that EF1a interacts with the viral RNA-dependent RNA polymerase and the 30-terminal genomic RNA of tobacco mosaic virus (TMV) during *Nicotiana benthamiana* infection [23]. Interestingly, knockdown of expression of the gene encoding EF1a inhibits the cell death response in soybean and alters this host response to soybean mosaic virus [24]. On the other hand, EF1b can disrupt EF1a-induced actin organization [25]. EF1b is also engaged in the growth and cell cycle regulation [26]. However, the roles of EF1a and EF1b’s in plant-RKN interactions have not been described yet.

The frontline of the plant defense system constitutes the cell wall, whose dry mass in 90% is composed of cellulose, hemicelluloses, and pectins [27]. Polygalacturonases (PGs) are known to be engaged in the last step of pectin degradation but their role in plant development has been also described [28]. Moreover, in maize, PGs have been reported to take part in the suppression of programmed cell death [29]. There are also some data indicating the importance of plant PG during *Glycine max* response to *Heterodera glycines* infection. It has been also shown that the upregulation of *PG* transcription in soybean roots in the early stage of nematode infection could facilitate successful parasitism [30]. However, most of the studies on plant-pathogen interactions have been focused on the role of pathogen’s PGs and plant polygalacturonase-inhibiting proteins (PGIPs) (e.g. [31]), while the function of plant’s PGs in defense response has not been very widely analyzed. On the other hand, the role of glycine-rich proteins (GRPs) was relatively well studied in the infection process. Among others, GRPs have been suggested to initiate the recognition of the stimuli from the environment and to participate in signal transduction [32]. GRPs have been also identified as part of the defense and repair system of the plants but their mode of action on the molecular level is still not clear [33]. Moreover, the large spectrum of subcellular locations and the broad structural diversity of GRPs suggests that they are involved in several independent physiological processes [34]. Besides the functions related to cell wall functioning and plant defense response, GRPs have been described to act as extracellular ligands of kinase proteins, RNA-binding proteins during osmotic stress, and many others [35]. The regulation of RNA metabolism by glycine-rich RNA-binding protein has been also reported as important for plant innate immunity [36]. The glycine-rich RNA-binding proteins are known to be involved in post-transcriptional regulation of gene expression as well as in RNA processing, which is part of developmental regulation in plants [37].

The study presented is aimed at characterization of gene expression of the following transcription and elongation factors: *WRKY53*, *EF1a*, and *EF1b* and the genes encoding two proteins related to cell wall functioning: glycine-rich RNA-binding protein 2 (*GRP2*) and polygalacturonase (*PG*), during the maize response to *M. arenaria* infection. For the study the transcription and elongation factors genes that have not been thoroughly analyzed yet, have been chosen. The relative changes in the expression level of the genes encoding these proteins were assessed using reverse transcription-quantitative real-time PCR (RT-qPCR) using RNA isolated from leaves and root samples derived from maize varieties showing different susceptibilities toward *M. arenaria* infection.

## Materials and methods

### Material

The experiments were conducted on maize plants from four varieties of different susceptibilities toward *M. arenaria* infection. Two sensitive varieties: PR38F58 (Pioneer) and Tasty Sweet (Seminis) as well as two tolerant varieties: PR39A98 (Pioneer) and Multitop (Syngenta) were selected during our previous study [7].

*Meloidogyne arenaria* larvae were collected from tobacco roots using NaOCl, according to the technique described by Hussey and Barker [38].

### Growing conditions and sample collection

The 3–4 week old seedlings of maize plants at the 4–5 leaves stage were inoculated with 1500 J2 stage larvae of *M. arenaria* suspended in water. From each variety, four infected and four non-infected control plants were grown at constant day/night temperatures of 25 °C/20 °C and under controlled light conditions. Samples from roots and leaves from healthy and nematode-inoculated plants were collected at three time points: 24 h (hpi), 3 days (dpi), and 7 days (dpi) after inoculation.

### RNA extraction and cDNA synthesis

From all leaves and roots samples, total RNA was extracted using Spectrum™ Plant Total RNA Kit (Sigma-Aldrich). RNA concentration and purity were determined with a NanoDrop 2000 spectrophotometer (Thermo Fisher Scientific). From each sample, 200 ng was used as a template for cDNA synthesis with a Maxima First Strand cDNA Synthesis Kit for RT-qPCR with dsDNase (Thermo Fisher Scientific). The cDNA has been diluted 1:1 with sterile distilled water and used as a template for the real-time PCR assay.

### Real-time PCR reactions

Real-time PCR reactions were carried out with primers amplifying the reference genes: *Leunig* and *FPGS* [39] as well as genes encoding transcription and elongation factors *WRKY53*, *EF1a*, and *EF1b* and the genes encoding proteins connected with plant cell wall: polygalacturonase (*PG*) and glycine-rich RNA-binding protein 2 (*GRP2*), using specific primer pairs listed in the table below (Table 1).

**Table 1:** Primers used in this study with their annealing temperatures and target genes

Target gene	GenBank number	Forward primer	Reverse primer	Annealing temperature (°C)
*Leunig* (reference)	NM_001158123	GTCAGGAACCCCAACCCTAT	CTCCCAACACCACCTTGATT	61
*FPGS* (reference)	NM_001350861	ATCTCGTTGGGGATGTCTTG	AGCACCGTTCAAATGTCTCC	61
*WRKY53*	KJ726810.1	CGCTCACCAAGGATCCCAAG	TGACGATGAAAGAACTGCTGC	60
*EF1a*	XM_008657932.3	CATGCTCTCCTTGCGTTCAC	CCATACCAGGCTTGATGACAC	60
*EF1b*	EU965401.1	CCTGGCGCTGAGTTTCCTAA	TTAGAAGAGGCCTTGGCAGC	60
*PG*	NM_001154783.2	CTCATTCACGGAGAGGGCTT	GTTTGGAGCATCCAGGGGAG	60
*GRP2*	EU963153.1	TTCGCTTCTGCTACCGTGTT	ATCGGTGGAGCTCAATGCAG	60

All subsequent qPCR reactions were conducted in three biological replicates and three technical replicates, in 10 µl of reaction mixture containing: 1 μl of template cDNA, 0.5 μM of each primer, 5 μl of iTaq master mix (Biorad), and sterile distilled water. Moreover, a sample with no cDNA template was used to exclude the reagents contamination. Reactions were performed using LightCycler 96 (Roche) with the following thermal profile: 5 min at 95 °C; 40 cycles at 95 °C for 10 s, annealing temperature appropriate for each primers pair (Table 1) for 10 s and 72 °C for 10 s. The melting phase began at 65 °C and ended at 95 °C, with an increase of 1 °C at each step. Relative quantification analyses were performed using the GenEx 6.0 software (MultiD Analyses AB) using the formula: Relative quantities = 2^−ΔΔCq^. Expression data were normalized using the genes encoding *Leunig* and *FPGS* as references [36]. Statistical significance of down- or up-regulation was also calculated with GenEx using the *t*-student test and *P* < 0.05.

## Results

The analysis presented in this study concerned the expression of genes of maize proteins from two groups: those encoding transcription and elongation factors (WRKY53, EF1a, and EF1b) and those related to plant cell wall (PG and GRP2), in the varieties of maize showing different susceptibilities to *M. arenaria*, at different time points after nematode infection.

### Changes in the expression level of genes encoding transcription and elongation factors

**Fig. 1:**
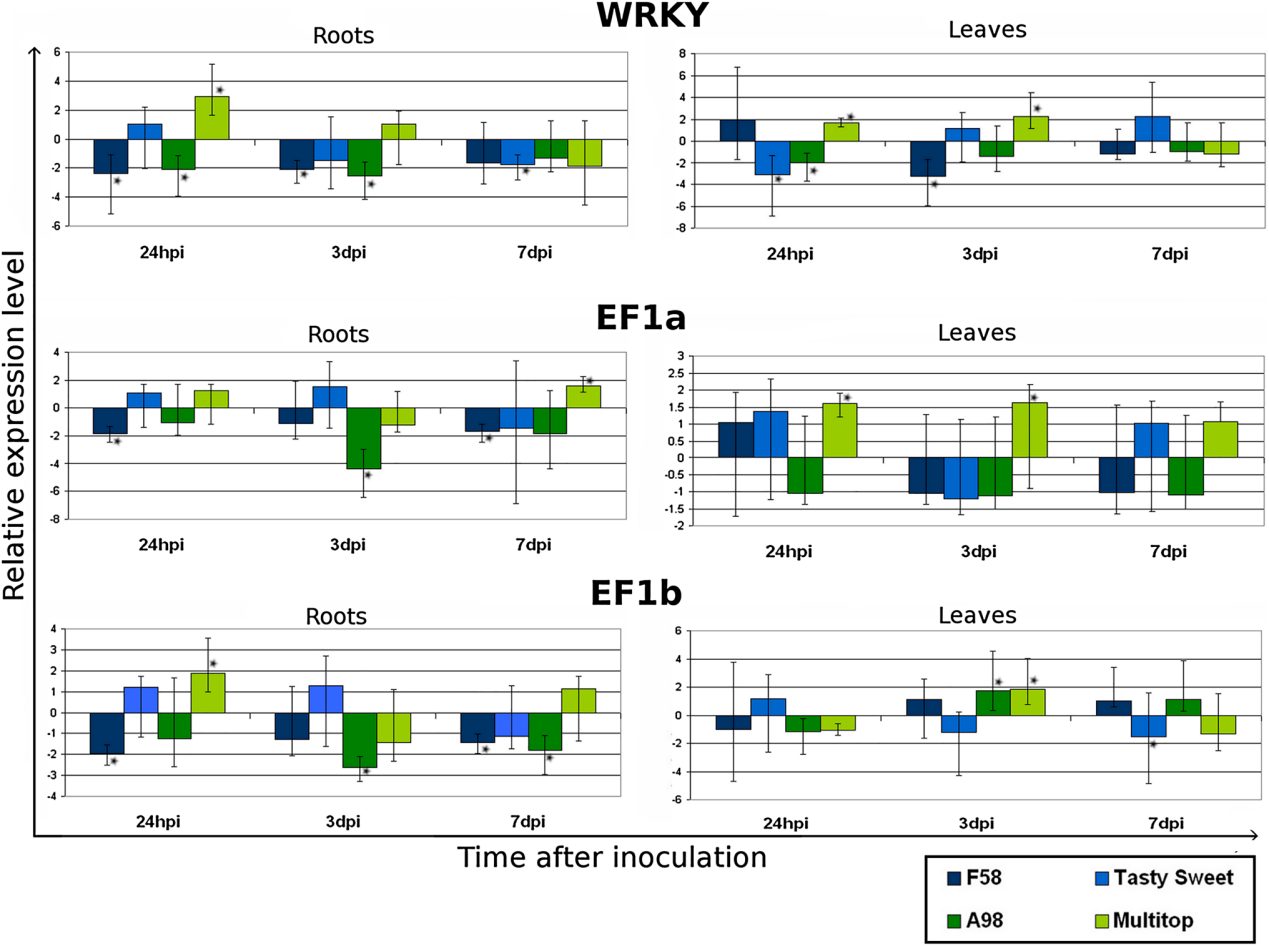
The relative expression level of genes encoding transcription and elongation factors: WRKY53, EF1a and EF1b. Significant down- or up-regulation (*P* < 0.05) is indicated with an asterisk

The analysis of the expression of genes encoding transcription and elongation factors revealed some changes between the samples taken at different time points after nematode inoculation as well between inoculated varieties. These differences were particularly pronounced in the level of expression of the gene encoding EF1b protein. In the samples collected from the roots, the lowest expression level was found 3 days after the infection, especially for one of the tolerant varieties—PR39A98. On the other hand, in the samples from the leaves collected from the tolerant varieties, a significant upregulation of *EF1b* gene expression was observed 3 dpi and was suppressed later 7 dpi. In the expression level of the gene encoding WRKY53, there were some changes between the samples collected at different time points but no significant changes between the sensitive and tolerant varieties nor between the samples from the roots and leaves were observed (Fig. 1).

### Changes in the expression level of genes encoding cell wall-related proteins

**Fig. 2:**
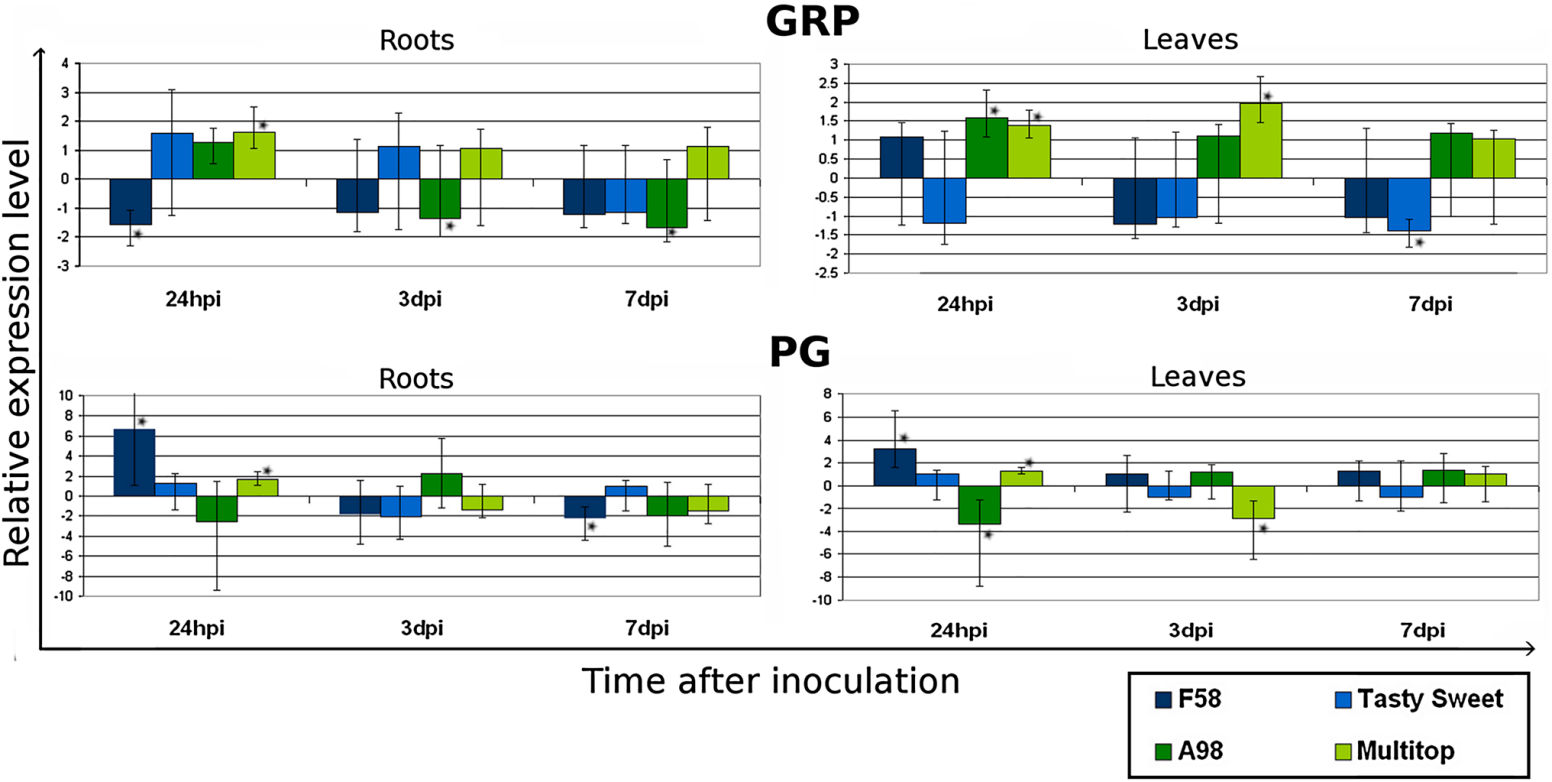
The analysis of the relative changes in the gene expression level of genes encoding proteins related to the cell wall: glycine-rich protein (GRP) and polygalacturonase (PG). Significant down- or up-regulation (*P* < 0.05) is indicated with an asterisk

Significant changes were found in the *GRP2* gene expression between the samples collected from the roots and leaves as well as those taken at different time points. For the samples from the roots the changes in the level of this gene expression appeared mainly between the samples collected at different time points. In most varieties, in the first stage of infection (24 hpi), the upregulation of *GRP* expression occured, while downregulation was observed only in the sensitive variety—PR39F58. In the later stage of infection, 7 dpi, the expression level decreased in all varieties except the tolerant one–Multitop. In this variety, the upregulation was observed in the samples collected at all time points. In the leaves a significant difference was noted between the results obtained for the sensitive and tolerant varieties. In both tolerant varieties, PR39A98 and Multitop, the upregulation of the *GRP* gene expression was observed at all time points tested, in contrast to sensitive varieties. For the latter, the upregulation was observed only at the very beginning of infection (24 hpi) for PR39F58 and then the gene expression was downregulated. In the second sensitive variety, Tasty Sweet, the gene expression was suppressed at all time points. On the other hand, significant upregulation of *PG* gene expression was observed for one sensitive variety (PR39F58) in the early stage of infection (Fig. 2).

## Discussion

Root-knot nematode infection causes numerous changes in many metabolic pathways in the host plant. Molecular mechanisms of plant-*Meloidogyne* interactions are still not fully understood. Many studies indicate an important role of transcription factors and cell wall-related proteins in the infection process and plant resistance to infection in dicotyledonous (e.g. [10, 40–43]) as well as in monocotyledonous hosts [8, 9, 11, 44].

In this study, we reported the upregulation of the expression level of the gene encoding WRKY53 transcription factor in one tolerant variety (Multitop) in the early stage of infection. This finding partially coincides with the results obtained for WRKY13 and WRKY24 transcription factors during interactions of RKNs and their monocotyledonous host—rice, in which the upregulation of the genes encoding these proteins was observed only in tolerant varieties in all stages of infection [8, 9, 11]. The WRKY53 protein analyzed in this study has been reported to be involved in plant response to pathogens, including bacteria [45], viruses [46], and herbivores [47] but there have been no data on its role in plant-RKNs interaction yet.

Previous analyses on tomato-*M. incognita* pathosystem have shown the downregulation of another gene—*EF1*—in the later stages of nematode infection in a susceptible variety of *Solanum lycopersicum* [40]. However, no data on the role of EF1a and EF1b proteins in the response of monocotyledonous to RKNs infection have been available. Our results indicated some significant changes in the expression level of gene encoding EF1b, which may suggest a role of this protein in the plant response and plant resistance to infection with *M. arenaria* species. In contrast to our results for biotic stress conditions, EF1a and EF1b have been described as stable genes in plants under abiotic stresses and are widely used as reference genes in qPCR reactions [48].

Two other proteins analyzed in this study are related to cell wall functioning. In our experiment the upregulation of the *PG* gene in the sensitive variety was observed, which corresponds with the data reported for *H. glycines*–*G. max* pathosystem, for which the upregulation of the gene encoding polygalacturonase was also observed during compatible interactions [30]. Another gene analyzed in our study was that encoding a protein belonging to GRPs family. These proteins are known to be scaffold or agglutinating agents for the deposition of cell wall constituents in plant's cell wall structure [35]. This proteins group is characterized by the presence of semi-repetitive glycine-rich motifs [34]. The role of GRPs in the response of many plant species to biotic and abiotic stress conditions has been described previously [32]. The downregulation of *GRPs* expression has also been observed during virus infection of tobacco [49]. Moreover, one of the glycine-rich RNA-binding proteins (AtGRP7) has been described to play either a positive or negative role in defense against different pathogens. AtGRP7 confers plant defense against a tobacco mosaic virus in tobacco as well as *Pectobacterium carotovorum* in *Arabidopsis*, but on the other hand, the same protein plays a negative role in defense against the fungus *Botrytis cincera* in *Arabidopsis* [50]. Additionally, the plant species displaying increased levels of GRP transcripts have been found more resistant to biotic and abiotic stress conditions than the wild-type plants [51, 52]. This observation is in agreement with the results obtained in our studies. During maize infection by *M. arenaria* we observed a significant upregulation of expression of the gene encoding a glycine-rich RNA-binding protein in the tolerant varieties at all time points. In our previous study of the same pathosystem, varieties, and time points we analyzed marker genes from JA- and SA-mediated pathways, *e.g. PR1*, *PR3*, *PR4, PR5*, or *LOX*. We observed a decrease in their expression level 24 hpi, followed by an increase in expression to the basal level 3 dpi for most of the analyzed genes associated with both, JA- and SA- pathways. Moreover, the downregulation of PR-3 and PR-4 at 24 hpi was more pronounced in the tolerant varieties than in the sensitive ones [7]. Another study of a rice-*M. graminicola* pathosystem has shown that JA- and SA-mediated pathways are activated during the early stage of infection in both susceptible and resistant hosts, but the responses are suppressed in later stages of infection in the susceptible varieties [8, 9]. According to these results, there is no direct correlation between the changes in *GRP* expression level and the expression level of marker genes related with JA- and SA- mediated pathways, because for *GRP* no suppression was observed and upregulation occurred at all analyzed time points.

## Conclusions

The results of our study indicated a potential role of transcription and elongation factors in maize response and resistance to *M. arenaria* infection. However, the crucial finding of our study is that the most significant differences between susceptible and tolerant varieties were observed in the expression level of the gene encoding the glycine-rich RNA-binding protein, which was substantially higher in the tolerant varieties at all time points tested. This result is in agreement with the other studies on the GRPs role in plant response to biotic and abiotic stress conditions.
